# A Preliminary Analysis of Circulating Tumor Microemboli from Breast Cancer Patients during Follow-Up Visits

**DOI:** 10.3390/curroncol31090421

**Published:** 2024-09-21

**Authors:** Hung-Chih Lai, Hsing-Hua Huang, Yun-Jie Hao, Hsin-Ling Lee, Chiao-Chan Wang, Thai-Yen Ling, Jen-Kuei Wu, Fan-Gang Tseng

**Affiliations:** 1 Division of Hematology and Oncology, Department of Internal Medicine, Shin-Kong Wu Ho-Su Memorial Hospital, Taipei 11101, Taiwan; ctpetlai@gmail.com; 2 Division of Breast Surgery Clinic, En Chu Kong Hospital, No. 258, Zhongshan Rd., Sanxia Dist., New Taipei City 237, Taiwan; h610129@gmail.com; 3 Department of Engineering and System Science, National Tsing Hua University, No. 101, Sec. 2, Kuang-Fu Rd., Hsinchu 30013, Taiwan; hyjtb2009@gmail.com (Y.-J.H.); sabrinalee0623@gmail.com (H.-L.L.); joy710101@gmail.com (C.-C.W.); 4 Graduate Institute of Pharmacology, National Taiwan University, No. 33, Linsen S. Rd., Zhongzheng Dist., Taipei City 100025, Taiwan; tyling@ntu.edu.tw; 5 Biomedical Science and Engineering Center, National Tsing Hua University, No. 101, Sec. 2, Kuang-Fu Rd., Hsinchu 30013, Taiwan; 6 Research Center for Applied Sciences, Academia Sinica, Taipei 115, No.28, Alley 70, Section 2, Academia Road, Nankang District, Taipei City 115201, Taiwan

**Keywords:** breast cancer, circulating tumor cells (CTC), circulating tumor microemboli (CTM), liquid biopsy biomarker, tumor immune microenvironment

## Abstract

Background: Most breast cancer-related deaths are caused by distant metastases and drug resistance. It is important to find appropriate biomarkers to monitor the disease and to predict patient responses after treatment early and accurately. Many studies have found that clustered circulating tumor cells, with more correlations with metastatic cancer and poor survival of patients than individual ones, are promising biomarkers. Methods: Eighty samples from eleven patients with breast cancer during follow-up visits were examined. By using a microfluidic chip and imaging system, the number of circulating tumor cells and microemboli (CTC/CTM) were counted to assess the distribution in stratified patients and the potential in predicting the disease condition of patients after treatments during follow-up visits. Specific components and subtypes of CTM were also preliminarily investigated. Results: Compared to CTC, CTM displayed a distinguishable distribution in stratified patients, having a better AUC value, in predicting the disease progression of breast cancer patients during follow-up visits in this study. Four subtypes were categorized from the identified CTM by considering different components. In combination with CEA and CA153, enumerated CTC and CTM from individual patients were applied to monitor the disease condition and patient response to the therapy during follow-up visits. Conclusions: The CTM and its subtypes are promising biomarkers and valuable tools for studying cancer metastasis and longitudinally monitoring cancer patients during follow-up visits.

## 1. Introduction

Breast cancer is the most diagnosed malignancy and the second leading cause of cancer-related death in females worldwide [1], partially due to its unusual preference for metastasis. Breast cancer is often found to metastasize to lung, bones, and the brain, resulting in unfavorable prognoses, and the recurrence rate in patients with middle- and late-stage breast cancer is still as high as 50% within three years after treatment [2]. One limiting factor in dealing with metastatic breast cancer is chemoresistance, leading to failed treatments and eventual patient deaths. Immunotherapy, which features the elimination of malignant tumors by the immune system of the host, and is less affected by genetic variations and has fewer side effects in patients after treatment, has increasingly gained attention as a cancer treatment [3]. But cancer cells may act dynamically toward immune recognition during the period of immune surveillance [4], resulting in the different fates of cancer cells, including some that may still be able to escape from the immune system and to replicate [5]. Hence, it is necessary to study the interaction and relationship between cancer cells and their surroundings.

As for breast cancer metastasis, it is a complex and heterogeneous process involving various interactions—anatomically, cellularly, and molecularly. For instance, breast cancer cells could gain direct access to the systemic circulation by invading veins in the node [6], and the mechanical factor may play an important role in sentinel lymph node metastasis [7]. So far, linear and parallel progression are two potential routes that have been discovered in the metastatic process, which may involve the circulating tumor cell (CTC) to a great extent. Before infiltrating the blood vessels, CTC may contain tumor cells undergoing the epithelial-to-mesenchymal transition (EMT) to facilitate the detachment from the primary tumor site. Some CTC may contain or be derived from cancer stem cells (CSCs), helping to induce new pathological sites after migration and resistance to treatment [8,9]. The number of CTC in the blood has been associated with disease progression in cancers [10,11,12], including metastatic breast cancer [13]. It has been observed that the circulating tumor microemboli (CTM) or CTC cluster could also be linked to the poor prognosis of cancer patients [14,15]. However, due to limitations in technology and the more unusual and friable characters of CTM compared to CTC, a few studies have investigated the CTM and preliminarily profiled the clustered CTC in the life of CTC in vivo [16]. Some studies have also observed that CTC clusters might coexist with individual CTC in peripheral blood during tumor dissemination and metastasis [17], and tumor cells tend to form CTM in the metastatic stage [18,19,20], having more responsibilities for distant malignant colonization and tumor recurrence [21,22] than solitary CTC [23]. Due to a more supportive microenvironment and the possibility of becoming physically trapped at capillaries, a potentiality offered by clustered CTC for extravasation [24], multicellular tumor cell aggregates have been thought to play a major role in metastasis compared to solitary cancer cells. The presence of CTM in patient blood has increasingly been seen as an indicator of patients with shorter survival periods [25,26]. With improvements in technology and the involvement of microfluidic systems, the composition and structure of clustered CTC and their impacts on cancer metastasis and patient survivals have gradually become the focus of studies [27]. However, on the one hand, CTC clusters are quite vulnerable to being preprocessed. On the other hand, it is difficult to distinguish the functional CTC cluster from others through current commercially available CTC detection systems [28,29]. 

To address these issues, we previously developed a microfluidic-based cell self-assembly array chip (SACA) to enable multiple rounds of fluid to be exchanged on monolayered cells with minimal loss [30], and a chip-based automatic imaging system (CytoSCM imaging@cellenvision, https://www.cellenvision.biz/, accessed on 18 September 2024) equipped with four fluorescents/filters, a monochrome light source, and a CMOS sensor (Sony Inc., Tokyo, Japan) to take high-content multichannel fluorescent images [31]. By using the SACA chip and chip-based imaging system in this paper, we investigated the CTC, and especially the heterotypic CTC clusters (CTM), in a group of breast cancer patients during their follow-up visits after treatment. After multichannel screening, four maximal fluorescent markers in combination with a bright field view of cell morphologies were applied to investigate the composition and expression of specific hormonal receptors in CTM in breast cancer patients. Based on the analysis of both cell surface markers and cell morphology, the identified CTM were further categorized into subtypes with different components. From our preliminary results, compared to CTC, the CTM with more distinguishable distribution in stratified patients could be a promising biomarker for monitoring breast cancer patients longitudinally during follow-up visits after treatment, and a valuable tool to study cancer metastasis.

## 2. Materials and Methods

### 2.1. Enrollment of Clinical Patients

Protocols for enrolling clinical patients with breast cancer in this study were performed and registered under the framework of the Institutional Review Board (IRB-number: 20171007R, 20180711R, 20190817R) of En Chu Kong Hospital and Shin Kong Wu Ho-Su Memorial Hospital. All patients agreed and signed their informed consent forms for the IRB. All patients were diagnosed with breast cancer at an early stage when they were recruited in this study during their follow-up visits from 20 January 2022 to 31 August 2023 at En Chu Kong Hospital and Shin Kong Wu Ho-Su Memorial Hospital. Chemotherapy (at least once) and/or hormone therapy were involved in their treatments, and they were sampled at every one of their follow-up visits. Every patient was followed-up at least three times.

### 2.2. Image-Based CTC/CTM Detection

#### 2.2.1. Plasma Preparation and Cell Enrichment

Plasma from peripheral vein blood samples of breast cancer patients were prepared, as described previously [32]. In brief, red blood cells were firstly removed from 2 mL whole blood samples kept in a collection tube with K2EDTA (BD Vacutainer^®^, Plymouth, UK), after being mixed with a Lymphopre^TM^ Density medium (STEMCELL technology, Vancouver, BC, Canada) in the Leucosep^TM^ separation tubes (Bio-Check Laboratories Ltd., New Taipei City, Taiwan) by centrifugation. Then, plasma and CTC/CTM-containing PBMCs were collected and transferred to another tube. After another centrifugation, CTC/CTM-enriched cell pellets were separated from the plasma, gently washed, and resuspended in Phosphate-buffered saline (PBS) (Corning, Corning, NY, USA) for further detection.

#### 2.2.2. Immunofluorescent Staining

Resuspended cells in PBS were counted and stained by Hoechst 33342 (Thermo Fisher Scientific Taiwan Co., Ltd., Taipei, Taiwan), anti-pan cytokeratin (PanCK) conjugated with fluorescein isothiocyanate (FITC) (Catalog number: IR222-861-FITC, iReal Biotechnology Inc., Hsinchu, Taiwan), anti-CD45-phycoerythrin (PE) (Catalog number: 12-0459-42, Thermo Fisher Scientific Taiwan Co., Ltd., Taipei, Taiwan), anti-CD3-APC (Catalog number: E-AB-F1001E), anti-CD8-APC (Catalog number: E-AB-F1110E), and anti-estrogen receptor alpha (ER) -PE (Catalog number: AB209288, Abcam, Blossom Biotechnologies Inc., Taipei, Taiwan) in the dark at room temperature to label the nucleus, CTC/CTM, leucocytes, lymphocytes, and the estrogen receptor (ER) on CTC/CTM. Then, cell suspension was carefully washed by PBS. 

#### 2.2.3. CTC/CTM Detection and Enumeration

**Figure 1:**
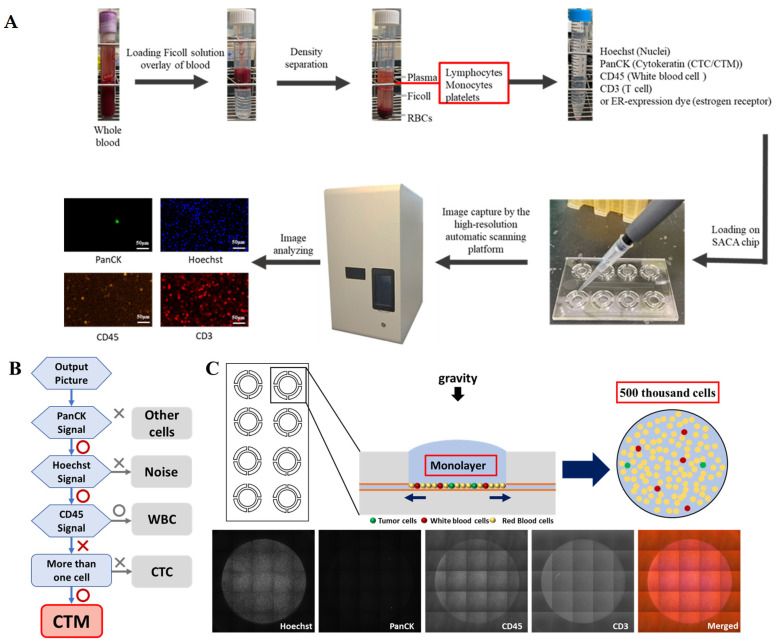
Flow chart of the workflow and schematic illustrations of the mechanisms and criteria to detect CTC/CTM in this study. (**A**): The workflow of the isolation and enumeration of CTC/CTM from blood samples by our SACA-chip-based imaging platform; (**B**): an overview of the workflow coded by the automatic scanning platform to take high-content image analysis for CTM identification; (**C**): schematic images of the working mechanism of the SACA chip and output images by the SACA-chip-based imaging platform. Details regarding the SACA-chip and chip-based imaging system can be found in the Supplementary Data.

After immunostaining, cell suspension was loaded onto the self-assembled cell array (SACA) chip previously developed in our group [30] and further examined by the chip-based automatic scanning platform (anti-estrogen receptor alpha-PE (Catalog number: AB209288, Abcam, Blossom Biotechnologies Inc., Taipei, Taiwan) (CytoSCM imaging@cellenvision) [31]. After scanning, high-resolution fluorescent images in different channels were obtained for analysis. By examining both the immunofluorescent signal and morphological data, individual and clustered CTC on the output image were identified and analyzed by the imaging system, which was already equipped with programs to collect information including the fluorescent signal and intensity, the aspect ratio, equivalent radius, eccentricity, and the roundness of the labelled cells and their 2D location and area on the chip. The PanCK+/Hoechst+/CD45− CTC and CTC clusters were finally identified and enumerated. The workflow flowchart and the mechanism of the chip are displayed in Figure 1. Details about the SACA chip and imaging system are provided in Supplementary Materials.

#### 2.2.4. Identification of the Subtypes of CTM with Potential Immune Microenvironment

Except for identifying and enumerating the CTC/CTM (PanCK+/Hoechst+/CD45−), the panel of PanCK/Hoechst/CD45/CD3 was used for analyzing the immune microenvironment of CTM, and the panel of PanCK/Hoechst/ER/CD8 was applied for further examining the ER expression and the potential immune microenvironment of CTM. Compared to individual CTC, the clustered CTC were further classified into four subtypes after examining the immune microenvironment and morphology of CTC in this study, including the CTC cluster with only PanCK+ CTC, the clustered CTC (PanCK+) accompanied by only CD45+ leukocytes in which the ratio of PanCK+ CTC and CD45+ leukocytes was less than 1, the small group of clustered CTC (PanCK+) accompanied by CD45+ leukocytes and CD3+ lymphocytes, in which the ratio of PanCK+ CTC and the sum of CD45+ leukocytes and CD3+ lymphocytes was 1 or more than 1, and the large group of clustered CTC (PanCK+) accompanied by CD45+ leukocytes and CD3+ lymphocytes, in which the ratio of PanCK+ CTC and the sum of CD 45+ leukocytes and CD 3+ lymphocytes was less than 1.

### 2.3. Statistical Analysis

Identification of CTC/CTM and subtypes were processed by the image processing program of the automatic scanning platform. The enumerated CTC/CTM were then analyzed using Microsoft^®^ Excel (Microsoft^®^ office 365 version 2019, Redmond, WA, USA). The threshold value of the enumerated CTC/CTM to predict the status of disease was identified by the Receiver Operating Characteristic (ROC) curve and the related area under the ROC Curve (AUC), processed by the GraphPad Prism 8 for Windows (GraphPad Software Inc., San Diego, CA, USA). The best cut-off point with the optimized sensitivity and specificity were also determined by the GraphPad Prism 8.

## 3. Results

### 3.1. The Design of This Study

**Figure 2:**
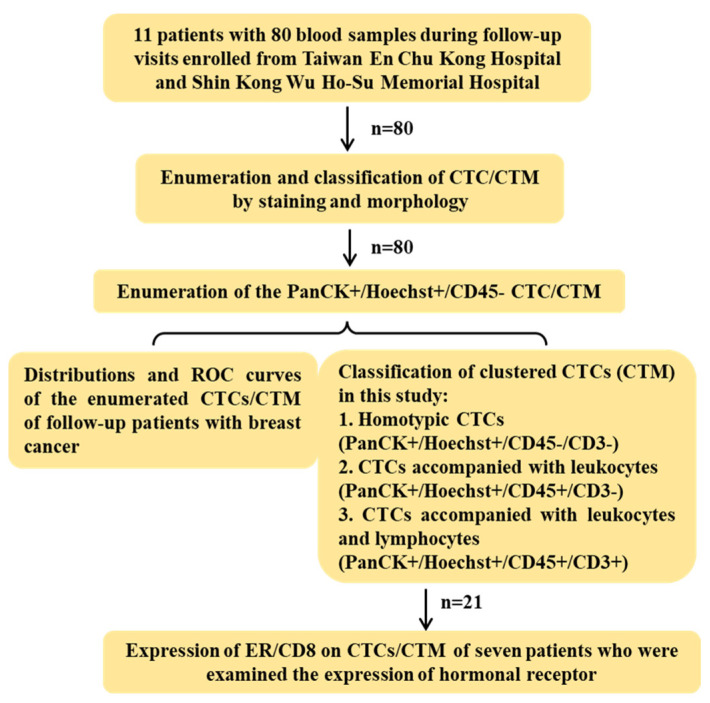
Design of this study. In total, 11 breast cancer patients with 80 blood samples during follow-up visits were enrolled from Taiwan En Chu Kong Hospital and Shin Kong Wu Ho-Su Memorial Hospital. Based on both immunofluorescent staining and morphology, CTC/CTM were firstly counted to study the distribution and ROC curves. The sample observing CTM further examined the immune microenvironment of CTM by staining the panel of markers with CD3 or CD8 to be classified subtypes. A total of 21 samples with detected CTM from seven patients were clinically examined for the expression of hormonal receptors, and the expression of estrogen receptors and CD8 were also preliminarily investigated in the detected CTM of patients after treatments during follow-up visits.

Eleven patients with breast cancer during their follow-up visits from 20 January 2022 to 31 August 2023 at En Chu Kong Hospital and Shin Kong Wu Ho-Su Memorial Hospital were enrolled and sampled at each visit for this study. All blood samples were then sent to the lab in the National Tsing Hua University for further processing and analysis within 24hrs. The design of this study was displayed in Figure 2. All 80 samples were examined for the number of CTC/CTM based on immunostaining and morphology. To assess the immune microenvironment of CTM, CD45 in combination with CD3 or CD8 in different panels of staining were examined to unveil potential components of immune cells such as leukocytes and lymphocytes. An anti-ER antibody was also used to investigate and monitor the expression of the estrogen receptor alpha (ER) on the identified CTC/CTM in seven patients with 21 samples in total (patients 3 to 9) after treatment during their follow-up visits.

### 3.2. The Demography of Patients with Breast Cancer Enrolled in This Study

All 11 patients (Table 1) were female and in middle age (44–62 years old). Except for the last one (stage IV), the other 10 patients were clinically diagnosed with early-stage breast cancer (0–II stage in the TNM stage) during their first few visits. Most of patients were suffering from left-breast cancer (8 out of 11), and two patients were confirmed as experiencing metastasis. All patients’ tissue biopsy samples were examined to check the expressions of the estrogen receptor, progesterone receptor, human epidermal growth factor receptor 2 (HER2), and the Ki67 (MKI67) value (Table 1), and two patients belonged to the triple-negative breast cancer (TNBC) group. Nine patients who were diagnosed as disease-free during follow-up visits in this study were from En Chu Kong Hospital. The other two patients were diagnosed with disease recurrence, of which one was from En Chu Kong Hospital and the other was from Shin Kong Wu Ho-Su Memorial Hospital. Patients were sampled at every one of their follow-up visits, and every patient was followed-up at least three times (Table 1).

### 3.3. Distribution and the Receiver-Operating Characteristic (ROC) Curves of CTC/CTM from Patients with Breast Cancer

**Figure 3:**
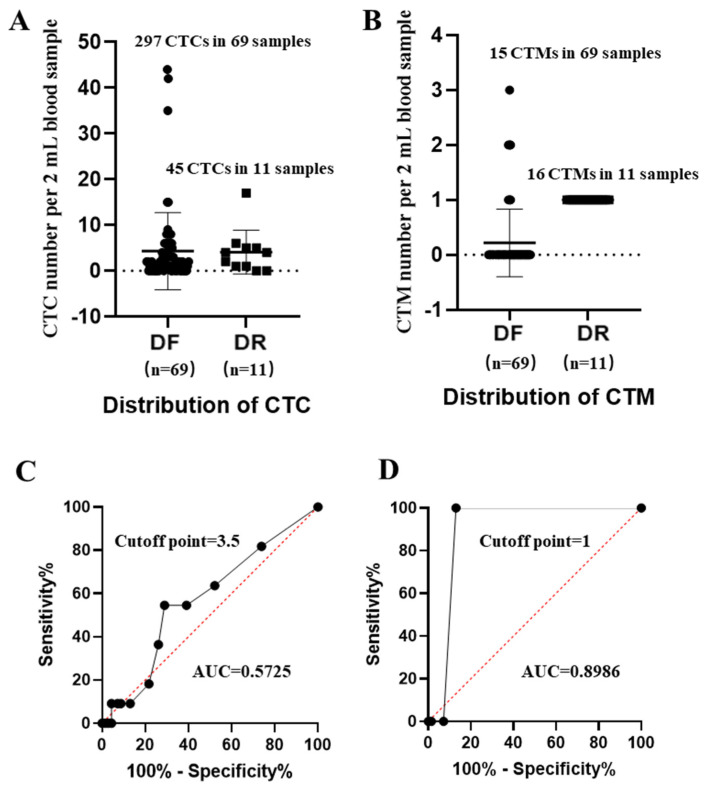
Distributions of CTC/CTM in stratified patients and the receiver-operating characteristic curves (ROCs) of CTC/CTM. (**A**): Distributions of CTC in patients diagnosed as disease-free (DF) and disease recurrence (DR); (**B**): distributions of CTM in patients diagnosed as DF and DR; (**C**): ROC curve and AUC value of CTC; (**D**): ROC curve and AUC value of CTM. The median CTC number per sample from the DF group was 4.3, and 4.09 from the DR group; the median CTM numbers per sample from the DF and DR groups were 0.2 and 1.45, respectively.

Based on enumerations (Supplementary Materials Table S2), distributions of CTC and CTM in stratified patients (Figure 3A,B) and ROC curves of CTC and CTM, respectively, were analyzed (Figure 3C,D).

Compared to CTM, distributions of CTC in disease-free (DF) and disease recurrence (DR) samples were more overlapped, but there was difficulty in distinguishing their individual abilities for predicting the state of disease, which were further confirmed by their individual ROC curves and the related AUC values. From results, cut-off points of CTC/CTM were 3.5 and 1, respectively. The corresponding AUC value of CTC was 0.5725 (*p* value = 0.4422), while that of CTM was 0.8986 (*p* value < 0.0001). Although the number of CTM (31 CTM in 80 samples) observed in this study was less than that of CTC (342 CTC in 80 samples), the predictive power of CTM was much stronger. We also observed that, in DF samples, the identified single CTM often contained fewer than two CTC, while in DR samples CTM was more frequently to be observed in 2 mL blood samples, and more than 2 CTC were more likely to be found inside one identified single CTM.

### 3.4. Characterization of CTM Subtypes

Based on previous microscopic observations showing the heterogeneity of our identified CTC in blood samples, details of CTC in terms of volume and shape of whole cells and organelles, nucleus to cytoplasm (NC) ratio, and dense nuclear materials to nucleus ratios were primarily analyzed from high-content images of the observed single CTC by the program developed for our automatic image scanning platform. 

**Figure 4:**
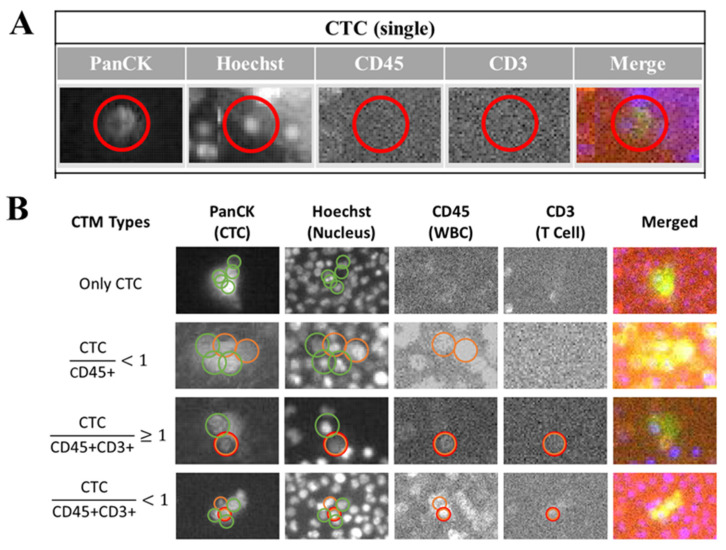
Classification of clustered CTC (CTM) by image-based analysis. After immunofluorescent staining to label-specific markers including the PanCK, Hoechst, CD45, and CD3 on CTC/CTM, the cell-loaded SACA-chips were scanned by the automatic imaging platform (CytoSCM imaging system). Digitally labeled images of CTC/CTM in different channels (PanCK: FITC channel; Hoechst 33342: UV365 channel; CD45: PE channel, CD3: APC channel, and Merged channel) were further analyzed to detect, enumerate, and identify different CTC subpopulations. (**A**): Output images of different channels to show the individual CTC identified by the platform; (**B**): output images of different channels to show four subtypes of clustered CTC identified by the platform, including the CTC cluster with only PanCK+ CTC, the clustered CTC (PanCK+) accompanied by only CD45+ leukocytes in which the ratio of PanCK+ CTC and CD45+ leukocytes was less than 1, the small group of clustered CTC (PanCK+) accompanied by CD45+ leukocytes and CD3+ lymphocytes, in which the ratio of PanCK+ CTC and the sum of CD45+ leukocytes and CD3+ lymphocytes was 1 or more than 1; the large group of clustered CTC (PanCK+) accompanied by CD45+ leukocytes and CD3+ lymphocytes, in which the ratio of PanCK+ CTC and the sum of CD 45+ leukocytes and CD 3+ lymphocytes was less than 1. The red circle in (**A**) was displayed to show the identified area of the targeted CTC in different channels by the automatic imaging platform. The green circle in (**B**) was displayed to show the identified CTC in different channels by the automatic imaging platform. The orange circle in (**B**) was displayed to show the identified leukocytes in different channels by the automatic imaging platform. The red circle in (**B**) was displayed to show the identified lymphocytes in different channels by the automatic imaging platform.

From the output images of samples (Figure 4), according to the fluorescent label and morphological information such as the cell surface texture, the circularity of cells and the NC ratio of cells, these identified CTC could be generally classified into individual CTC (Figure 4A) and clustered CTC (Figure 4B). The clustered CTC could be further divided into four groups in this study, including a homotypic CTC cluster (formed by PanCK+ CTC only) and a heterogenous CTC cluster (CTM). Considering different immune microenvironments around CTC in a cluster, CTM could be then divided into CTC (more than two CTC) accompanied with leukocytes in a ratio lower than one (PanCK+ CTC/CD45+ < 1), a small group of CTC (one or two CTC) accompanied by both leukocytes and lymphocytes in a ratio of 1 or more than one (PanCK+ CTC/CD45+ and CD3+ ≥ 1), and a large group of CTC (more than two CTC) accompanied by both leukocytes and lymphocytes in a ratio lower than 1 (PanCK+ CTC/CD45+ and CD3+ < 1).

### 3.5. Patient Management by Monitoring Liquid Biopsy Biomarkers CTC/CTM

**Figure 5:**
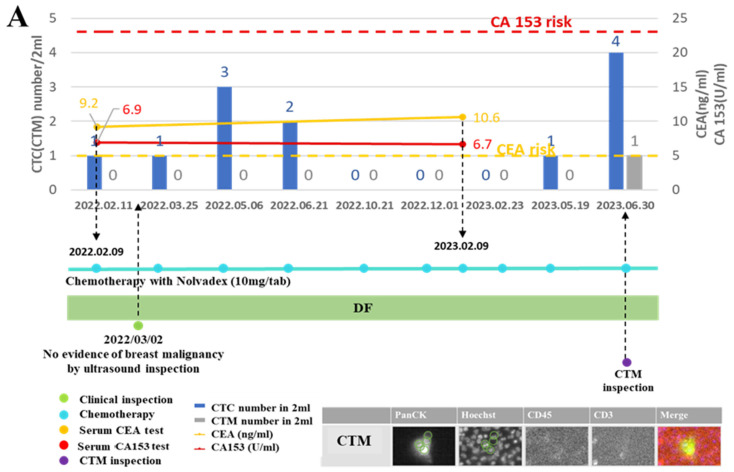

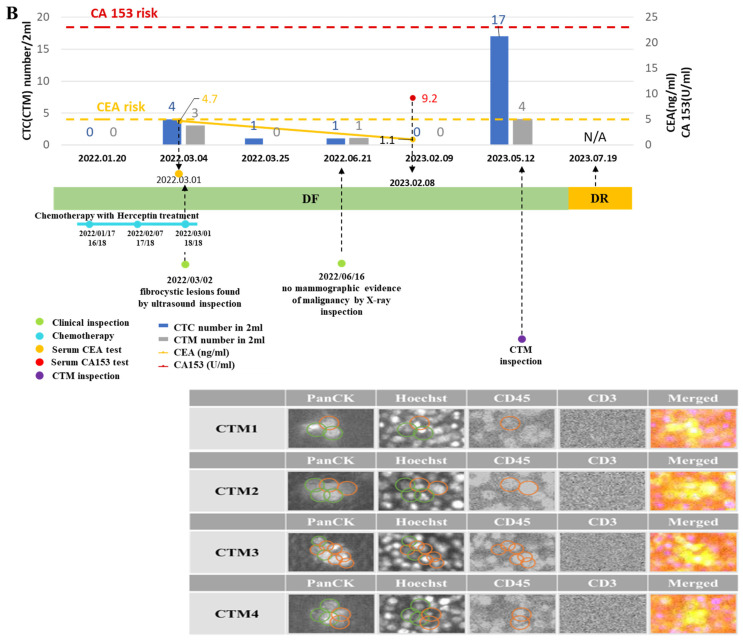
Monitoring curves of liquid biopsy biomarkers from two exemplified breast cancer patients diagnosed with disease-free and disease recurrence, respectively. (**A**): Monitoring curves of liquid biopsy biomarkers from the patient diagnosed as disease-free (DF) during follow-up visits; (**B**): monitoring curves of liquid biopsy biomarkers from the patient diagnosed with disease recurrence (DR) during follow-up visits. The x–axis is the time line of a series of follow-up visits of the patient, and the y–axis is used to display the enumerated CTC/CTM at each follow-up visit accompanied by the clinical data of CA 153 level and CEA level at the specific checkpoint during follow-up visits. The red dotted line shows the CA153 risk line, and the yellow dotted line shows the CEA risk line on the curve. The number of CTC/CTM are displayed in histograms in blue and gray, respectively, on the curve. The blue line dotted at each follow-up visit represents the chemotherapy and drugs applied to the patient. The green dot is used to show the clinical inspection examined at that visit. The purple dot is used to show the CTM analysis accompanied by the output images to identify the CTM. CTM1 to CTM4 denoted four CTM observed from the sample collected from this patient at the follow-up visit (12 May 2023). The green circle in CTM images was displayed to show the identified area of the targeted CTC in different channels by the automatic imaging platform. The orange circle in CTM images was displayed to show the identified leukocytes in different channels by the automatic imaging platform.

By monitoring a series of liquid biopsy biomarkers including serum levels of cancer antigen 15-3 (CA 15-3) and carcinoembryonic antigen (CEA), the CTC/CTM of patients with breast cancer during their follow-up visits, the state of disease, patient responses to the chemotherapy, and antitumor drugs could be clearly demonstrated. Two exemplified monitoring curves of one disease-free (DF) patient (Figure 5A) and one disease recurrence (DR) patient (Figure 5B) are displayed in Figure 5.

From the monitoring curve of the patient diagnosed with DF (Figure 5A), her CEA level was higher than the risk, but other markers were kept at lower risk throughout the period of the chemotherapy treatment with antitumor drugs. At one follow-up visit in the beginning, there was no evidence of breast malignancy observed by ultrasound inspection, while at the last visit a CTM was observed. From further imaging analysis of this identified CTM, it belonged to a homotypic CTC cluster involving only CTC (no leukocytes or lymphocytes).

From the monitoring curve of the patient diagnosed with DF (Figure 5B) during follow-up visits, those biomarkers were all generally kept at lower levels and the patient was not diagnosed with DR until the last visit (20230719). There were no fibrocystic lesions found by ultrasound inspection and no mammographic evidence of malignancy by X-ray inspection during the first few follow-up visits when this patient was continuously diagnosed with DR during these visits. However, at the visit two months (20230512) before the last one, both CTC and CTM presented in high-risk levels, in which the number of CTC was 17 and that of CTM was 4 (counted from a 2 mL blood sample). From the CTM inspection, it was further observed that CTM from this patient at this visit were heterogenous, containing more than two CTC accompanied by CD45+ cells but no CD3+ cells. Different immune components might be relevant to the condition of the disease of the patient. 

### 3.6. ER Expression in CTC/CTM of Breast Cancer Patients with Treatment

**Figure 6:**
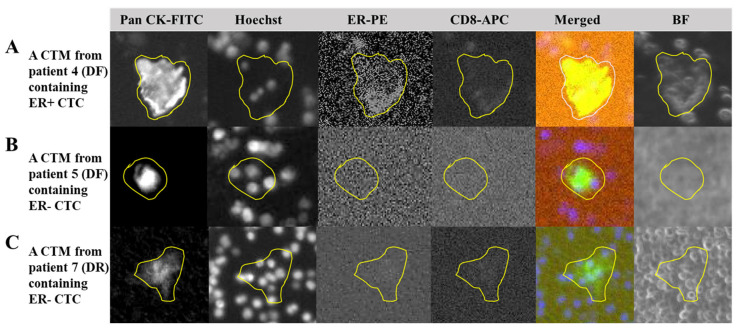
Expressions of the estrogen receptor and CD8 in the CTM identified in breast cancer patients. (**A**): Output images from four channels of our chip-based imaging system together with the merged and bright field images to illustrate the PanCK+ CTC, nuclei, ER expression (positive), and CD8 expression (positive) in the CTM identified from patient 4 who was diagnosed as disease-free (DF) during follow-up visits; (**B**): output images from four channels of our chip-based imaging system together with the merged and bright field images to illustrate the PanCK+ CTC, nuclei, ER expression (negative), and CD8 expression (negative) in the CTM identified in patient 5 who was also diagnosed as disease-free (DF) during follow-up visits; (**C**) output images from four channels of our chip-based imaging system together with the merged and bright field images to illustrate the PanCK+ CTC, nuclei, ER expression (negative), and CD8 expression (negative) in the CTM identified in patient 7 who was experiencing hormonal therapy and diagnosed with disease recurrence (DR) during follow-up visits. After immunofluorescent staining to label-specific markers including the PanCK, Hoechst, and CD45 to identify the CTM, samples were also examined for the expression of CD8 and ER on the identified CTM. The cell-loaded SACA-chip was scanned by the automatic imaging platform CytoSCM. The digitally labeled area of the identified target in images from four different channels (PanCK-FITC; Hoechst 33342-UV; ER-PE; CD8-APC), merged images, and bright field images are circled by the yellow line.

Through immunostaining, the expression of estrogen receptor alpha (ER) was preliminarily monitored in CTM identified from seven patients (n = 21) who showed the expressions of ER/PR/HER2 in their clinical records examined. In most DF samples, the expressions of ER in the identified CTM were negative (Figure 6B), as observed in images from patient 5 who was diagnosed as DF, showing a positive expression of ER in her clinical record (Table 1). In the CTM of patient 7 who was diagnosed as DR and showing a negative expression of ER from her clinical record (Table 1) consistently, there was no ER expression observed (Figure 6C). However, we did observe the positive expression of ER (Figure 6A) in the CTM of patient 4 who was also diagnosed as DF, showing the positive expression of ER from her clinical record (Table 1). In addition, it was found that the expression of CD8 was also positive in the identified PanCK+ CTM of patient 4, but negative in patients 5 and 7. The expression of the hormonal receptor in the CTC/CTM of breast cancer patients after clinical treatment might be variable during follow-up visits, suggesting the potential correlation of the expression of the hormonal receptor with clinical treatments.

## 4. Discussion

The liquid biopsy technique provides a convenient way to study such biomarkers as the circulating cell-free (tumor) DNA (cfDNA/ctDNA), circulating tumor cells (CTC), circulating tumor microemboli (CTM), tumor-related extracellular vesicles, and to analyze expressions of both sensitized and resistant genes in the bodily liquids of patients directly [33,34]. As biomarkers, both CTC and CTM have been increasingly investigated in clinical cancer studies. It has been observed that the number of CTC might be an independent factor associated with the prognosis of disease progression and patient response to treatment [8,9,35,36], and in most poorly controlled studies of patients, including metastatic and early-stage cancer cases, an elevated level of CTC/CTM numbers in patients’ blood may portend metastasis [37] and poor prognosis [38]. However, their clinical values are not fully validated and, in some cases, yield prejudiced results [39,40,41]. In a preclinical study, it was found that the number of CTC was insufficient for predicting patient response and stratifying breast cancer patients with neoadjuvant chemotherapy [42]. In this study, we also observed differences between the distribution of CTC and that of CTM in stratified patients, and when applying the number of CTC individually to analyze the ROC curve to predict the condition of disease in patients during follow-up visits, its predictive power was less fulfilled than that of CTM. 

As a type of rare cell, CTC-related studies usually require specific devices, most of which are image-based approaches relying on stained markers such as the FDA-approved CELLSEARCH, which isolates and enumerates the EpCAM+ (epithelial cell adhesion molecule) CTC from leukocytes and other epithelial non-tumor cells [43]. Furthermore, methods combining phenotype staining with various morphological criteria have emerged, involving size-based detection by the ScreenCell^®^ Cyto [44] or by the CellSieve™ microfilter [45], the observations of apoptosis-characterized nuclei [45,46], the specific nuclear morphology with a large nuclear-cytoplasmic (NC) ratio [47] or malignant nuclei [45], morphological identification of tumor cells [48], a series of cytomorphological analyses of the nuclei, cytoplasm, NC ratio, cell membrane and proliferation of cells [49], biosensor-based morphological detection [50], and so on. Although different methods have different limitations in terms of technique and areas of application, multichannel labelling/staining-based image processing with high-performing antibodies/dyes and double exclusion methods combined with supplementations from molecular and morphological analyses, would be a promising way to reduce potential false positives and maximize the true positive detection of functional CTC/CTM [51].

CTM were previously defined as a CTC cluster [52]. Then, more studies realized their complexity and demonstrated both in vitro and in vivo that clustered CTC were formed due to expressions of specific components in cell–cell junctions [27], containing more heterogenous components than single CTC [52]. Surroundings of CTC in the cluster, including different subtypes of CTC [13,53,54] and different cell–cell interactions [55], might determine and reflect the important role played by the clustered CTC in clinical settings [56,57]. It might be reasonable to define the CTM as a CTC cluster with specific components. For homotypic CTM made of only CTC, variations of CTC in clusters (such as undifferentiated vs. differentiated CTC, and epithelial CTC vs. epithelial-mesenchymal transition CTC) may make different contributions to collective invasion and cellular colonization at distant sites [58,59]. For heterotypic CTM consisting of CTC and other components (e.g., cancer-related stromal cells, immune cells, and platelets), the specific surroundings of CTC inside the cluster involving co-operation and crosstalk between different components may also facilitate CTC in the life circle and metastatic mission, such as to evade inspection of the immune system in the early dissemination, to survive in circulation, to extravasate at a targeted site, and to be activated genetically to restart proliferations in vivo [58,60].

In this study, by considering both cell surface markers and cell morphologies, we not only identified CTC and CTM, but also visualized and classified subtypes of the CTM, based on specific components regarding immune cells (immunofluorescent staining CD45/CD3/CD8) around CTC (Pan CK) inside the CTM. Our results confirmed the heterogeneity of clustered CTC and began expanding our previous knowledge of CTC and CTM. As displayed in Figure 4, four subtypes of CTM were identified in this study by our platform, including the CTM containing only PanCK+ CTC, the CTM containing PanCK+ CTC accompanied by only CD45+ leukocytes in which the ratio of PanCK+ CTC and CD45+ leukocytes was less than 1, the CTM containing a small group of clustered PanCK+ CTC accompanied by CD45+ leukocytes and CD3+ lymphocytes, in which the ratio of PanCK+ CTC and the sum of CD45+ leukocytes and CD3+ lymphocytes was 1 or more than 1, and the CTM containing a large group of clustered PanCK+ CTC accompanied by CD45+ leukocytes and CD3+ lymphocytes, in which the ratio of PanCK+ CTC and the sum of CD 45+ leukocytes and CD 3+ lymphocytes was less than 1. We believe that different subtypes of the CTM identified in the patient samples may have specific correlations with the condition of disease of the patient at the time of clinical inspection. From the personalized monitoring curves exemplified in Figure 5, we have included the analyzed images of the identified CTM. But due to the rarity of CTM in blood samples, the scale of samples, and the period of follow-up currently involved in this study, correlations of the subtypes of CTM with clinical outcomes should be improved by increasing the number of recruited patients and follow-up visits. In addition, the types of cell surface marker should be expanded to identify more subtypes of CTC (such as the epithelial-mesenchymal transition CTC, hormonal receptor + CTC) and further different components from CD45+ leukocyte and CD3/CD8 lymphocytes (such as NK cell, platelet, fibroblast, and macrophage) in our future works.

We preliminarily monitored the expression of ER on our identified CTC/CTM by our chip-based imaging platform. In breast cancer, though the ER expression on the tumor often correlates with the clinical decision around hormonal therapies, the expression level of ER in vivo is variable and the definition of the expression level of the ER should comply with the latest ASCO (the American Society of Clinical Oncology)/CAP (the College of American Pathologists) guidance [61]. Moreover, it was assumed that the development of distant metastases in women with ER-positive primary tumors during or after the endocrine therapy might be relevant to the surviving ER-negative CTC. This kind of survival could be explained either by the heterogeneity of primary tumors, releasing both ER-positive and ER-negative CTC into circulation, or by a switched ER expression due to genomic and/or epigenomic changes inside the in vivo microenvironment [62,63]. After all, a switch from the positive to negative ER expression might be one of the mechanisms for CTC to evade hormonal therapies [63,64,65]. Hence, it might be worth monitoring the expression of a hormonal receptor such as the ER in the identified CTC/CTM from breast cancer patients with treatment. These results would provide the real-time information for physicians to manage the response of patients to ongoing chemotherapy or hormonal therapy.

From our preliminary results (Figure 6), we observed the consistent expressions of ER in the identified CTM from patient 7 with a negative expression of ER in her clinical records, though she was diagnosed as DR at the last follow-up visit in this study. We also created the personalized monitoring curve for patient 7 (Figure 5B), when she was receiving Hercetine (10 mg/tab) treatment for three months (shorter than the usual of 8–11 months) and was unable to control the disease after 18-month follow-ups. Although a consistent negative expression of ER in her CTM with clinical records was observed, her CTM became more complicated (Figure 5B), suggesting potential correlations between CTM subtypes and the clinical outcome. Compared to the personalized monitoring curve of patient 7, in another curve for patient 3 (Figure 5A), there was a long period of follow-ups for this patient, and diagnoses of her condition of disease were kept as disease-free, consistent with our prediction from the CTM inspection, in which there was a simple composition in the CTM identified in her sample. We also observed dynamics of the expression of ER in samples from patients (patient 4 and 5) who had positive expressions of ER in clinical records and were both diagnosed as DF during follow-up visits. A consistent positive expression of ER was detected in patient 4 (Figure 6A), but a negative expression of ER (Figure 6B) was found in patient 5 during their follow-up visits, respectively. The dynamic of ER expressions may reflect the efficiency of chemotherapy or hormonal therapy for the patient during follow-ups, providing valuable information for physicians to adjust the treatment. Interestingly, we also found that the expression of CD8 was positive when the ER expression was positive in the CTM of patient 4, and it was negative when the ER expression was negative in the CTM of patient 7 or patient 5. The variable expressions of ER in the CTM from patients might be relevant to a specific immune microenvironment inside the CTM, worth further investigation in our future work.

Although most samples examined in this study were from patients diagnosed as disease-free (69 samples from 9 patients), and the number of samples from patients diagnosed with disease recurrence should be increased in future work from our preliminary results, expressions of the ER in the CTM from patients with breast cancer during follow-up visits could be variable and inconsistent with examinations in original records. The ER might not be a reliable, but is a necessary, marker in monitoring patients with breast cancer longitudinally. However, when we looked over the CTM in the monitoring curve, correlations between the changes in the number and complexity of the CTM, and the prognoses for the conditions of disease of patients with breast cancer during follow-up visits (Figure 5), could be clearly observed, suggesting that the CTM might be an appropriate biomarker for physicians to longitudinally monitor breast cancer patients during disease management. In addition, it might be worth examining the complexity of CTM further, especially particular components, to identify the subtype of CTM and its correlation with clinical outcomes of patients in the biomarker-based monitoring curves. These future works will help us to further clarify the biogenesis, configuration, and clinical function of the CTM and provide valuable information for physicians to make decisions about the subsequent treatment of cancer patients.

## 5. Conclusions

By considering both cell surface markers and cell morphology in this study, the rare CTC/CTM were detected and enumerated, and the CTM was further classified into four subtypes regarding specific components by using the SACA chip and chip-based imaging system. Compared to CTC, the CTM presented a more distinguishable distribution in stratified patients and a better AUC value in predicting the disease condition of patients with breast cancer during follow-up visits after treatment in this study. The CTM and subtypes of CTM with specific components might be valuable tools and promising biomarkers to be used to investigate the biogenesis of CTC/CTM and the potential mechanism of cancer metastasis extensively, and to be further applied to monitor the condition of disease in individual cancer patients after treatment and during follow-up visits.

## Figures and Tables

**Table 1:** List of 11 patients with breast cancer enrolled in this study.

Patient Number	Age	TNM Stage	ER/PR/HER2 Expression	Ki-67 Value	Breast Cancer Diagnosed at the First Visit	Condition of Disease during Follow-Up Visits	Number of Follow-Up Visits	Hospital
1	50	IIA	+/+/−	50%	Left breast cancer, invasive ductal carcinoma	disease free	11	En Chu Kong
2	45	IIA	−/−/−	90%	Left breast cancer, malignant neoplasm of unspecified site	disease free	7	En Chu Kong
3	62	IA	+/+/−	5%	Left breast cancer, intraductal carcinoma in situ	disease free	9	En Chu Kong
4	48	IA	+/+/−	30%	Right breast cancer, malignant neoplasm of unspecified site	disease free	3	En Chu Kong
5	47	IIA	+/+/−	30%	Left breast cancer, malignant neoplasm of unspecified site	disease free	10	En Chu Kong
6	48	IIA	+/+/+	50%	Right breast cancer, malignant neoplasm of unspecified site	disease free	8	En Chu Kong
7	59	IA	−/−/+	60%	Left breast cancer, invasive ductal carcinoma, positive resection margin	disease recurrence	7	En Chu Kong
8	48	IIA	+/+/+	80%	Left breast cancer, invasive ductal carcinoma, metastatic liver, luminal B	disease free	6	En Chu Kong
9	51	IIA	+/+/−	20%	Right breast cancer, malignant neoplasm of unspecified site, luminal B	disease free	7	En Chu Kong
10	49	IIB	−/+/+	20%	Left breast cancer, malignant neoplasm of unspecified site	disease free	8	En Chu Kong
11	44	IV	−/−/−	80%	Left breast cancer, invasive ductal carcinoma, metastatic bilateral lung	disease recurrence	8	Shin Kong Wu Ho-Su

+: positive expression; −: negative expression.

## Data Availability

The original contributions presented in the study are included in the article/Supplementary Materials, and further inquiries can be directed to the corresponding authors.

## Supplementary Materials

The following supporting information can be downloaded at: https://www.mdpi.com/article/10.3390/curroncol31090421/s1, Figure S1: The SACA chip; Figure S2: The detection mechanism using the SACA-chip; Figure S3: Simulation using the SACA chip; Figure S4: Schematic images of the SACA chip and mechanism; Figure S5: Automatic imaging machine and software procedure; Figure S6: Process flow of in situ CTC detection, capturing, and culturing; Figure S7: The SACA-chip-based automatic imaging system; Figure S8: CTC’s detection of five patients from stage IV colon cancer; Figure S9: The graph represents the comparison of CTC’s detection capabilities between the Digi-SACA chip and IsoFlux; Table S1: The table describes the comparison between the Digi-SACA chip and IsoFlux for the detection of CTC. Table S2: Enumerated CTC/CTM from all breast cancer patients in this study. References [30,31,66] are cited in the Supplementary Materials.
