# Supplementary material for: A Preliminary Analysis of Circulating Tumor Microemboli from Breast Cancer Patients during Follow-Up Visits
Source: Curr Oncol. 2024 Sep 21;31(9):5677–93. doi: 10.3390/curroncol31090421 (PMC11431662; doi:10.3390/curroncol31090421)
Supplement: Supplementary file 1 [file curroncol-31-00421-s001.zip › curroncol-3128552-supplementary.pdf]

Supplementary Materials

A Preliminary Analysis of Circulating Tumor Microemboli from Breast Cancer Patients during Follow-Up Visits

Hung-Chih Lai [1], Hsing-Hua Huang [2], Yun-Jie Hao [3], Hsin-Ling Lee [3], Chiao-Chan Wang [3], Thai-Yen Ling [4], Jen-Kuei Wu [3,5,*] and Fan-Gang Tseng [3,5,6,*]

1 Division of Hematology and Oncology, Department of Internal Medicine, Shin-Kong Wu Ho-Su Memorial Hospital, Taipei 11101, Taiwan; ctpetlai@gmail.com
2 Division of Breast Surgery Clinic, En Chu Kong Hospital, No. 258, Zhongshan Rd., Sanxia Dist., New Taipei City 237, Taiwan; h610129@gmail.com
3 Department of Engineering and System Science, National Tsing Hua University, No. 101, Sec. 2, Kuang-Fu Rd., Hsinchu 30013, Taiwan; hyjtb2009@gmail.com (Y.-J.H.); sabrinalee0623@gmail.com (H.-L.L.); joy710101@gmail.com (C.-C.W.)
4 Graduate Institute of Pharmacology, National Taiwan University, No. 33, Linsen S. Rd., Zhongzheng Dist., Taipei City, 100025 Taiwan; tyling@ntu.edu.tw
5 Biomedical Science and Engineering Center, National Tsing Hua University, No. 101, Sec. 2, Kuang-Fu Rd., Hsinchu 30013, Taiwan
6 Research Center for Applied Sciences, Academia Sinica, Taipei 115, No.28, Alley 70, Section 2, Academia Road, Nankang District, Taipei City 115201, Taiwan
* Correspondence: d927111@gmail.com (J.-K.W.); fangang@ess.nthu.edu.tw (F.-G.T.)

A few of previous data regarding the design and development of the SACA chip and the automatic imaging system, applied in this study, were supplemented below.

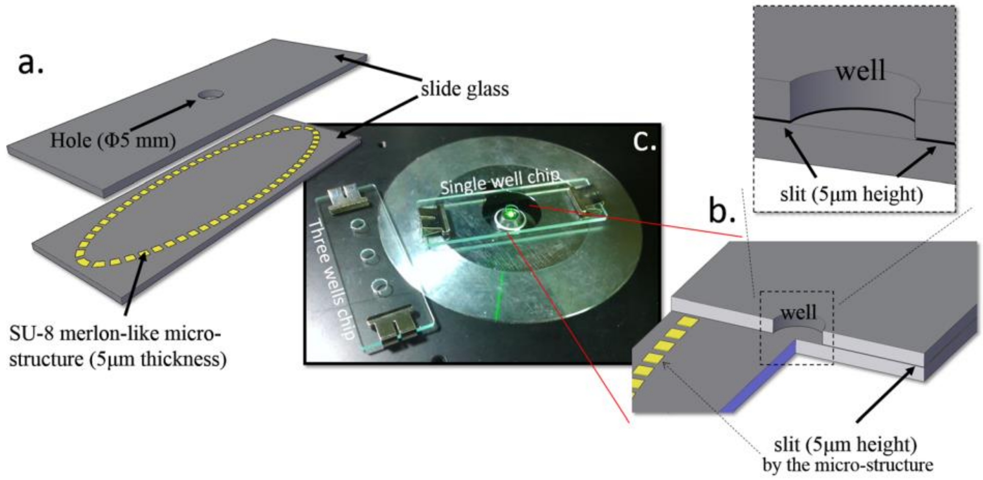

Supplementary Figure S1. The SACA chip. (a) Component schematic diagram. (b) The positional relationship of the well and the micro slit channel (chip sectional view). (c) The actual photos of chips on the inverted fluorescent microscope [1].

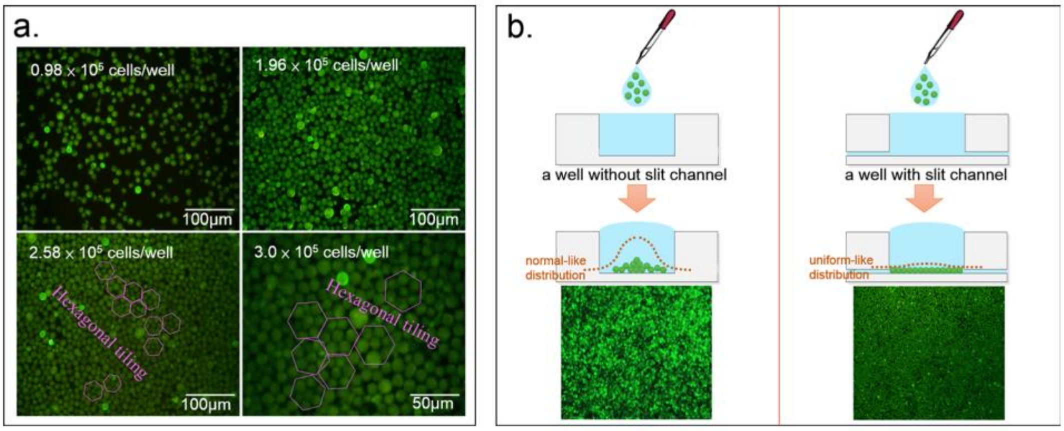

Supplementary Figure S2. (a) Different numbers of cells in the well leads to different types of cell organization. When the cell number exceeds 258 000 in the well, the cell arrangement begins to form hexagonal tiling, the highest density in 2D arrangement. (b) The bottom slit channel has a significant impact to the organization of the cell arrangement. It allows the cells to be distributed more evenly [1].

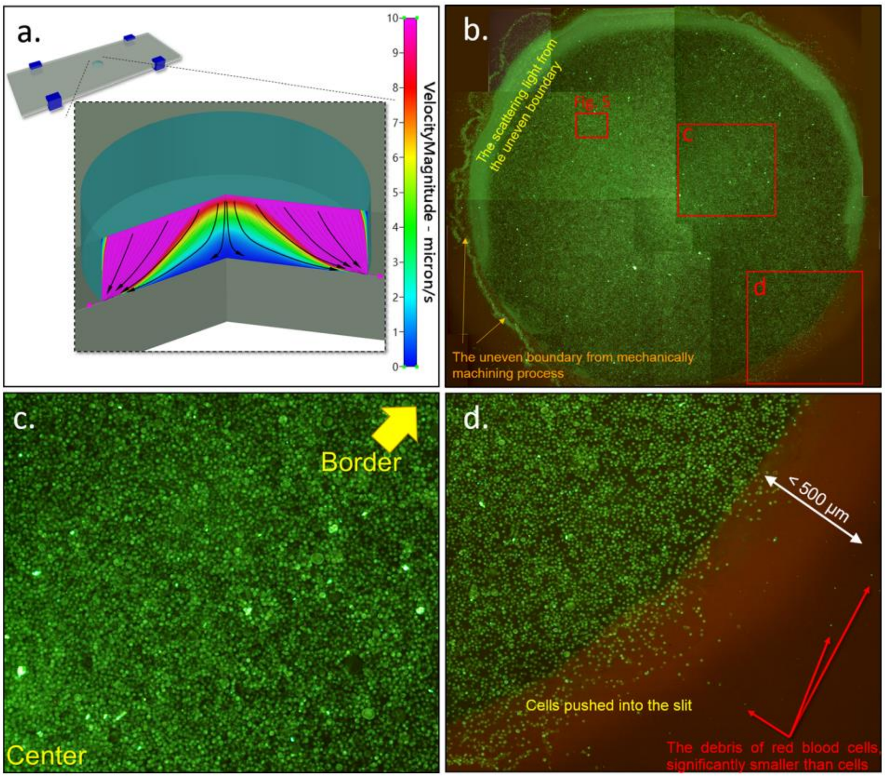

Supplementary Figure S3. (a) Numerical simulation results by CFD-RC illustrating the flow field in the self-assembly cell chip when the velocity of slip flow is 10 lm/s. The results show that the lateral velocity is higher when it is closer to the top or edge of the well. (b) Photograph of the cell arrangement in the well (diameter of 5 mm). The distribution of cells is similar to the results of numerical simulation. About 250 000 cells, 12 photos combined. (c) The region near center. (d) The border region [1].

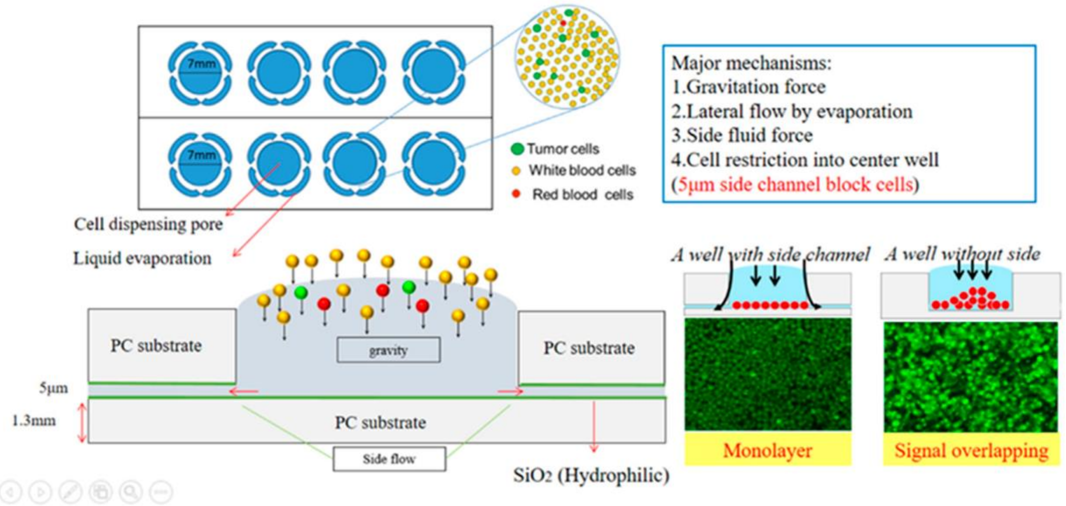

Supplementary Figure S4. The SACA chip consists of 8 wells with a 5 μm gap on the well bottom for cell restriction during the self-assemble process [2].

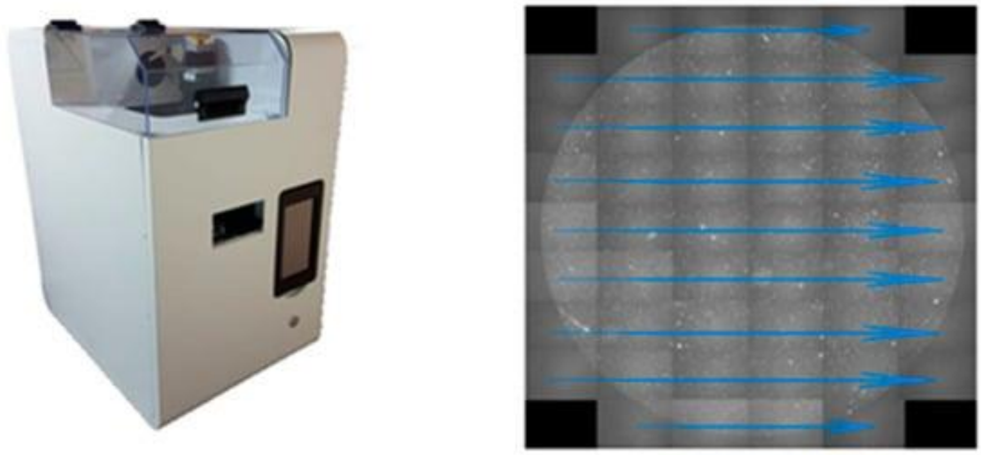

Supplementary Figure S5. Automatic imaging machine and software procedure [2]

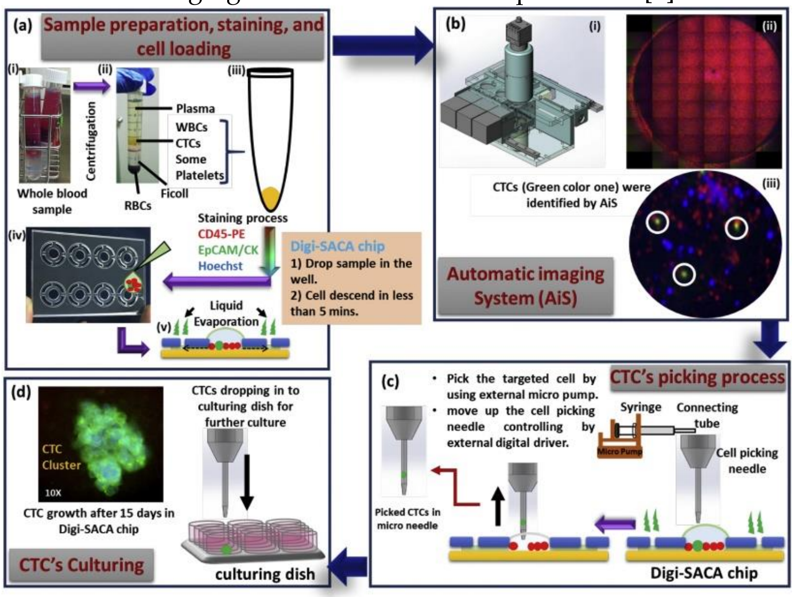

Supplementary Figure S6. Process flow of in situ CTCs detection, capturing, and its culturing. (a) Sample preparation and staining process. (b) AiS system to scan whole 7 mm well to detect CTCs. (c) CTC picking process. (d) Two types of CTCs culturing, in 96 well culture dish and direct culture in Digi-SACA chip [3].

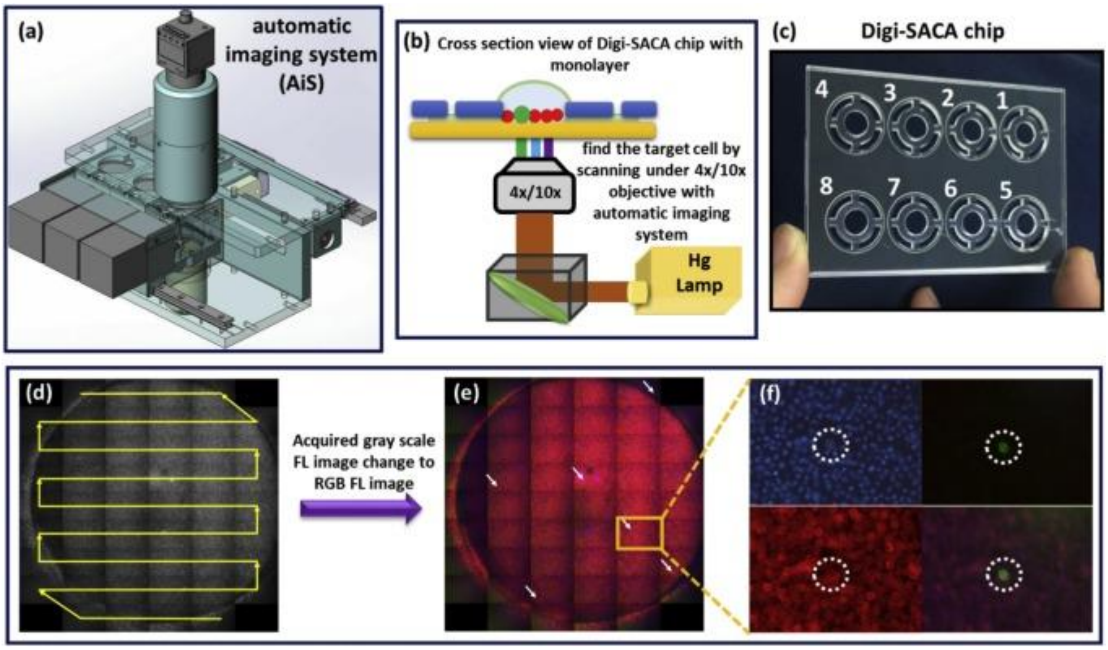

Supplementary Figure S7. Automatic imaging system. Automatic image system with CCD camera (a), Cross section view of Digi-SACA chip with cell monolayer and AiS with Hg lamp to scan all three fluorescence signals (b), The 8 wells in Digi-SACA chip were numbered to help in scanning (c), By selecting particular well in cell needle software, the AiS stage will move automatically to that particular well to scan, imaging and scanning direction fallowed the direction as in image (d), After scanning, gray scale stitched images have been changed to particular fluorescent color by using cell needle software as in (e), The single magnified image with CTC out of 50 stitched images (f) [3].

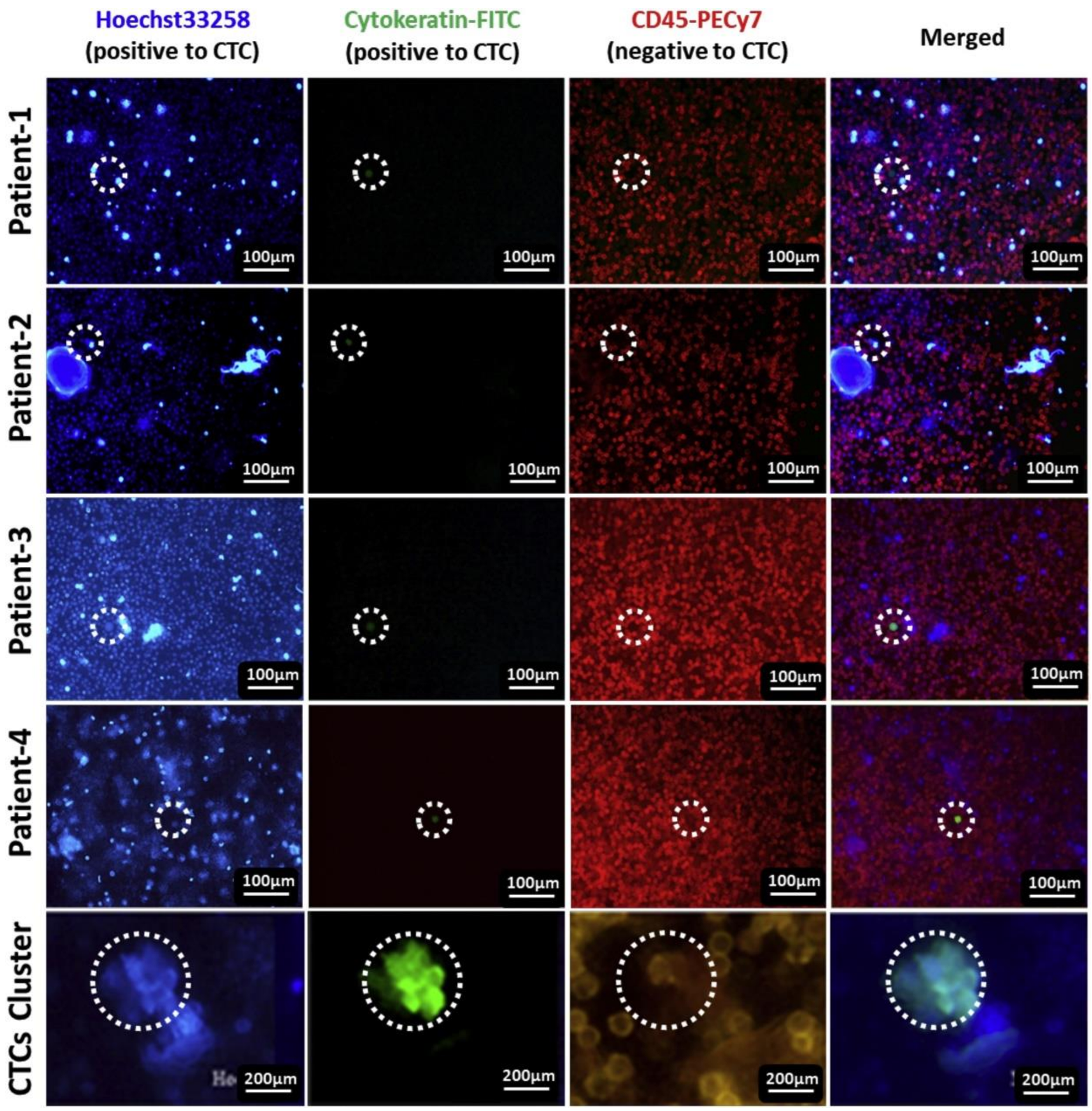

Supplementary Figure S8. CTC's detection of five patients from IV stage colon cancer. The fluorescent images show CTCs can be detected in high density without interference by 3 fluorescence signals. Hoechst33258 stains the cell's nucleus, cytokeratin-FITC stains CTCs' membrane and CD45-PE stains white blood cell membrane [3].

Supplementary Table S1. The table describes the comparison between Digi-SACA chip and IsoFlux for detection of CTCs from 10 samples (5 patients). The values represent the number of CTC's present in 4 ml blood sample [3].

Sample serial number	Patient ID (Total five patients)	IsoFlux (CTC number/4 mL)	Digi-SACA chip (CTC number/4 mL)
**1**	001	52	48
**2**	001	28	4
**3**	001	4	8
**4**	003	52	4
**5**	002	8	12
**6**	004	8	4
**7**	005	4	12
**8**	002	4	8
**9**	005	12	4
**10**	004	4	40

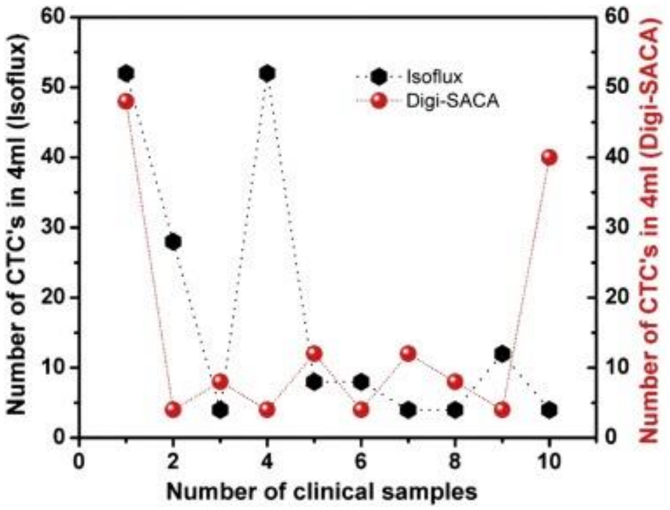

Supplementary Figure S9. The graph represents the comparison of CTC's detection capabilities between Digi-SACA chip and IsoFlux of 10 samples from 5 patents [3].

Details can be found in References below:

[1] Chen TJ, Wu JK, Chang YC, Fu CY, Wang TP, Lin CY, Chang HY, Chieng CC, Tzeng CY, Tseng FG. High-efficiency rare cell identification on a high-density self-assembled cell arrangement chip. Biomicrofluidics. 2014 May 1;8(3).

[2] Chu HY, Yang CY, Yeh PH, Hsu CJ, Chang LW, Chan WJ, Lin CP, Lyu YY, Wu WC, Lee CW, Wu JK. Highly correlated recurrence prognosis in patients with metastatic colorectal cancer by synergistic consideration of circulating tumor cells/microemboli and tumor Markers CEA/CA19-9. Cells. 2021 May 10;10(5):1149.

[3] Goudar VS, Yeh PH, Wu SY, Chu CH, Lu LS, Yang CH, Chiou TJ, Tseng FG. Live circulating tumour cells selection on digitized self-assembled cell array (Digi-saca) chip by in-parallel/in-situ image analysis, cell capture, and cultivation. Sensors and Actuators B: Chemical. 2020 Aug 1;316:128002.

Supplementary Table S2: Enumerated CTCs/CTM from all breast cancer patients in this study.

Patient number	Total number of CTCs/CTMs detected per 2ml analyzed blood											Diagnosed state of disease
1	1/0	0/0	0/0	2/0	0/0	0/0	3/0	0/0	0/0	0/0	1/0	Disease free
2	1/0	0/0	0/0	1/0	0/0	1/0	2/0					Disease free
3	1/0	1/0	3/0	2/0	0/0	0/0	0/0	1/0	4/1			Disease free
4	0/0	2/0	4/0									Disease free
5	1/0	0/0	8/0	35/0	2/0	3/0	6/1	15/1	5/0	3/2		Disease free
6	1/0	1/0	8/0	6/2	2/0	1/0	15/2	5/1				Disease free
7	0/0	4/3	1/0	1/1	0/0	17/4	NA/NA					Disease recurrence
8	0/0	44/2	6/0	1/0	0/0	1/0						Disease free
9	42/0	5/0	9/0	2/0	2/0	8/3	2/0					Disease free
10	1/0	3/0	3/0	6/0	3/0	6/0	0/0	6/0				Disease free
11	4/2	5/3	NA/NA	NA/NA	5/1	6/2	2/0	NA/NA				Disease recurrence

* NA denotes the failed detection of CTC/CTM in samples of this follow-up visit.
